# Exploratory Association of 5′ and 3′ UTRs Variants in *TYMS* and *CCND1* with Severe Hematologic Toxicity in Pediatric Acute Lymphoblastic Leukemia

**DOI:** 10.3390/ijms27188131

**Published:** 2026-09-12

**Authors:** Daniela Rojo-Serrato, Francisco Javier Flores-Murrieta, Haydeé Rosas-Vargas, Elva Jiménez-Hernández, Juan Carlos Núñez-Enríquez, Carolina González-Torres, Javier Gaytan-Cervantes, Deyanira Escalante-Bautista, María Luisa Pérez-Saldivar, Janet Flores-Lujano, América Mariana Jasso-Mata, Abimael Efraín Marquez-Águilar, José Ángel Sotelo Hernández, Omar Alejandro Sepúlveda-Robles, María de los Ángeles Romero-Tlalolini, Carmen Alaez-Verson, Jorge Alfonso Martín-Trejo, Silvia Jiménez-Morales, José Luis Cruz-Jaramillo, Norma A. Oviedo de Anda, Manuel A. Castillo-Mendéz, José Refugio Torres-Nava, Luz Victoria Flores-Villegas, Laura Elizabeth Merino-Pasaye, María Raquel Miranda-Madrazo, María de Lourdes Gutiérrez-Rivera, Karina Anatasia Solís-Labastida, Gabriela Alicia Herández-Echaurregui, Jorge Melendez-Zajgla, Angélica Rangel-López, José Arellano-Galindo, Minerva Mata-Rocha, Juan Manuel Mejía-Aranguré

**Affiliations:** 1 Escuela Superior de Medicina, Instituto Politécnico Nacional, Ciudad de México 11340, Mexico; danielarojo.serrato@gmail.com (D.R.-S.); fjfloresmurrieta@yahoo.com (F.J.F.-M.); 2 UIM en Genética Humana, Hospital de Pediatría, Centro Médico Nacional Siglo XXI, Instituto Mexicano del Seguro Social, Ciudad de México 06600, Mexico; hayrov@gmail.com (H.R.-V.); deyusdeyus@gmail.com (D.E.-B.); americajassomata@gmail.com (A.M.J.-M.); abimaelmarquez@gmail.com (A.E.M.-Á.); angelsotelo235@gmail.com (J.Á.S.H.); sero__82@hotmail.com (O.A.S.-R.); 3 Laboratorio de Farmacología Clínica y Experimental, Instituto Nacional de Enfermedades Respiratorias, Ciudad de México 14080, Mexico; 4 Servicio de Oncohematología Pediátrica, Hospital Pediátrico Moctezuma, Secretaria de Salud, Ciudad de México 15530, Mexico; elvajimenez@yahoo.com (E.J.-H.); torresoncoped@live.com.mx (J.R.T.-N.); gher8601@hotmail.com (G.A.H.-E.); 5 División de Investigación en Salud, UMAE Hospital de Pediatría, Centro Médico Nacional Siglo XXI, Instituto Mexicano del Seguro Social, Ciudad de México 06600, Mexico; jcarlos_nu@hotmail.com; 6 Laboratorio de Secuenciación, División de Desarrollo de la Investigación, Centro Médico Nacional Siglo XXI, Instituto Mexicano del Seguro Social, Ciudad de México 06600, Mexico; gonzaleztorrescaro@gmail.com (C.G.-T.); javier_gc50@hotmail.com (J.G.-C.); 7 UIM en Epidemiología Clínica, Hospital de Pediatría, Centro Médico Nacional Siglo XXI, Instituto Mexicano del Seguro Social, Ciudad de México 06600, Mexico; maria_luisa_2000_mx@yahoo.com (M.L.P.-S.); janetflores22@yahoo.com.mx (J.F.-L.); 8 Facultad de Medicina y Cirugía, UABJ (Universidad Autónoma Benito Juárez de Oaxaca), Oaxaca 68120, Mexico; romerotlalolini@gmail.com; 9 Laboratorio de Diagnóstico Genómico, INMEGEN (Instituto Nacional de Medicina Genómica), Ciudad de México 14610, Mexico; calaez@inmegen.gob.mx; 10 Servicio de Hematología, Hospital de Pediatría, Centro Médico Nacional Siglo XXI, Instituto Mexicano del Seguro Social, Ciudad de México 06600, Mexico; jorge.martin.trejo@gmail.com (J.A.M.-T.); kas_anastacia@yahoo.com (K.A.S.-L.); 11 Laboratorio de Innovación y Medicina de Precisión, INMEGEN (Instituto Nacional de Medicina Genómica), Ciudad de México 14610, Mexico; sjimenez@inmegen.gob.mx; 12 Departamento de Bioinformática y Tecnologías, Solaria Biodata, Ciudad de México 03100, Mexico; 13 Unidad de Investigación Médica en Inmunología e Infectología, Hospital de Infectología, Centro Médico Nacional La Raza, Instituto Mexicano del Seguro Social, Ciudad de México 06600, Mexico; naoviedoa@yahoo.com.mx; 14 Academia de Nutrición, Plantel Cuautepec, Colegio de Ciencias y Humanidades, Universidad Autónoma de la Ciudad de México, La Palma, Ciudad de México 07160, Mexico; manuel.castillo@uacm.edu.mx; 15 Servicio de Hematología Pediátrica, Centro Médico Nacional 20 de Noviembre, Instituto de Seguridad y Servicios Sociales de los Trabajadores del Estado, Ciudad de México 06350, Mexico; victoriabanco@yahoo.com.mx (L.V.F.-V.); sketch0712@gmail.com (L.E.M.-P.); guadalupe.mirandan@imss.gob.mx (M.R.M.-M.); 16 Servicio de Oncología Pediátrica, Hospital de Pediatría, Centro Médico Nacional Siglo XXI, Instituto Mexicano del Seguro Social, Ciudad de México 06600, Mexico; lourdes.gutierrez@imss.gob.mx; 17 Laboratorio de Genómica Funcional del Cáncer, Instituto Nacional de Medicina Genómica, Ciudad de México 14610, Mexico; 18 Unidad de Investigación en Enfermedades Infecciosas, Hospital Infantil de México, Secretaria de Salud, Ciudad de México 06720, Mexico; 19 Secretaría de Ciencia, Humanidades, Tecnología e Innovación Secihti, Ciudad de México 03940, Mexico

**Keywords:** methotrexate, hematologic toxicity, *CCND1* gene, *TYMS* gene, pharmacogenomics

## Abstract

Mexico has one of the highest incidences of childhood acute lymphoblastic leukemia worldwide and also reports one of the lowest 5-year overall survival rates; one cause is elevated treatment-related mortality from severe hematologic toxicity, a critical side effect during chemotherapy. Although association studies have identified common genetic variants linked to toxicity, the Mexican population remains incompletely explored. We performed targeted next-generation sequencing of genes involved in methotrexate and mercaptopurine metabolism in 96 Mexican children with ALL, stratified by hematologic toxicity severity according to CTCAE v5.0. Logistic regression revealed nominally significant associations between the TSER*3/TSER*2 genotype (rs45445694) in the 5′ UTR of *TYMS* severe hematologic toxicity (overdominant model: OR = 3.21; 95% CI = 1.23–8.38; *p* = 0.014 and codominant model: OR = 3.31; 95% CI = 1.17–9.34; *p* = 0.047). Additionally, the *CCND1* LD block (rs678653/rs7177) also showed nominal associations (overdominant model: OR = 2.60; 95% CI = 1.01–6.71; *p* = 0.044). None of these associations remained statistically significant after Bonferroni correction for multiple testing (corrected α = 0.000588). Patients with severe toxicity had lower 5-year overall survival than those without severe toxicity (70.2% vs. 90.5%; *p* = 0.0609), although the difference was not statistically significant. Variants in 5′ and 3′ UTRs regions were markedly enriched compared to coding sequences, underscoring the importance of regulatory region analysis.

## 1. Introduction

Leukemia is a malignant neoplasm of hematopoietic system origin characterized by hyperproliferation of leukocyte cells [1]. Acute lymphoblastic leukemia (ALL) is the most common type of neoplasia in the pediatric population. In Mexico, the age-standardized incidence rate (ASIRw) for pediatric ALL is 53.1 per million in Mexico City, one of the first reported worldwide above 50. Similarly, high rates persist in marginalized populations. In Puebla, the rate is 53.2; in Tlaxcala, 54.7; and in Oaxaca, 47.7 [2,3]. The five-year survival rate is low; a 2018 international study reported it at 53% in Mexico versus more than 90% in other countries [4]. The goal of treatment for ALL is to eradicate residual leukemic cells, and antineoplastic agents are associated with serious adverse effects. The induction phase, typically spanning the first 4–6 weeks of treatment, relies on multi-agent chemotherapy designed to eradicate leukemic blasts from the bone marrow. However, these agents cause profound, obligatory myelosuppression, leading to severe neutropenia and thrombocytopenia [5,6,7]. In relation to toxicity in pediatric ALL, Mexican cohorts have reported a mortality rate of 7.1% directly attributable to chemotherapy toxicity and an induction mortality rate of 5.3% (including toxicity, infections, and bleeding) [5,8]. This proportion is substantially higher than that reported in reference centers in the United States or Western Europe, where treatment-related mortality (TRM) typically falls below 2–4% [7,9]. These treatment-related deaths represent a decisive obstacle to therapeutic success and are largely preventable. Interindividual response to chemotherapeutics, including toxicity, is influenced by germline genetic variants in genes encoding transporters, metabolizing enzymes, and drug targets and is a discipline known as genomic medicine or personalized medicine. Genomic medicine uses DNA sequence variations to guide targeted therapy in accordance with the genetic profile of each individual. It implements strategies that incorporate genetic testing to predict and avoid drug-induced adverse effects while also optimizing therapeutic efficacy through personalized dose selection [10,11,12]. In ALL treatment, methotrexate and mercaptopurine form the backbone of the consolidation and maintenance phases; multiple pharmacogenetic studies have identified common polymorphisms in genes, such as *SLCO1B1*, *MTHFR*, or *TPMT*, associated with an increased risk of adverse effects [13,14,15]. However, these findings have shown inconsistencies across populations, and the fraction of toxicity variability explained by these common variants is limited. This suggests the involvement of less common or population-specific variants, particularly in populations with a unique genetic architecture such as the Mexican population. In this study, we used targeted next-generation sequencing (NGS) of the coding and 5′ and 3′ UTRs regions of genes in the methotrexate and mercaptopurine metabolic pathway in a cohort of Mexican pediatric patients with ALL. Our aim is to explore variants that might confer susceptibility to severe hematologic toxicity and to focus on the identification of new biomarkers in the Mexican population.

## 2. Results

### 2.1. Clinical Samples

This study included 96 pediatric patients diagnosed with ALL, with 52 males (54%); the median age was 8 years. Based on the risk classification established by the National Cancer Institute (NCI), 46 patients (48%) were in the high-risk group. Regarding treatment regimens, 68% of patients received the St. Jude Total Therapy XIII-B, and 32% received the BFM therapy. Recurrent rearrangements—t(9;22), t(12;21), t(1;19), and t(4;11)—were detected by RT-PCR in 85 patients (89%), as previously described [16]. The results showed that 2.3% had *ETV6::RUNX1* fusion, 5.8% had *TCF3::PBX1* fusion, and 3.5% had *BCR::ABL1* p190 fusion. The high-grade (severe) hematologic toxicity group (Grades 3–4) included 62 patients (64.5%), and the low-grade (mild) hematologic toxicity group (Grades 0–2) included 34 patients (35.4%). The details of specific events that were submitted for classification are presented in Supplementary Table S1. Grade 3–4 neutropenia was the most frequent event, affecting 62 patients (100% of patients with severe toxicity), followed by thrombocytopenia (58/62, 93.5%) and anemia (49/62, 79.0%). Transfusion requirements were common: 55 patients (88.7%) required transfusions, 48 (77.4%) required platelet transfusions, and 42 (67.7%) required RBC transfusions.

### 2.2. Hematotoxicity and Oncologic Outcomes

The high-grade (severe) hematologic toxicity group (Grades 3–4) included 62 patients (64.5%), and the low-grade (mild) hematologic toxicity group (Grades 0–2) included 34 patients (35.4%). The details of specific events are presented in Supplementary Table S1. Grade 3–4 neutropenia was the most frequent event, affecting 62 patients (100% of patients with severe toxicity), followed by thrombocytopenia (58/62, 93.5%) and anemia (49/62, 79.0%). Transfusion requirements were common: 55 patients (88.7%) required transfusions, 48 (77.4%) required platelet transfusions, and 42 (67.7%) required RBC transfusions. Twelve patients (19.4% of the severe toxicity group and 12.5% of the total cohort) died from causes directly attributable to treatment toxicity. The analysis of the timing of toxicity occurrence showed that severe toxicity occurs in both phases—100% of patients with severe hematological toxicity had events in the early phase, while 35.5% of them also had events during maintenance (Supplementary Table S1).

**Figure 1:**
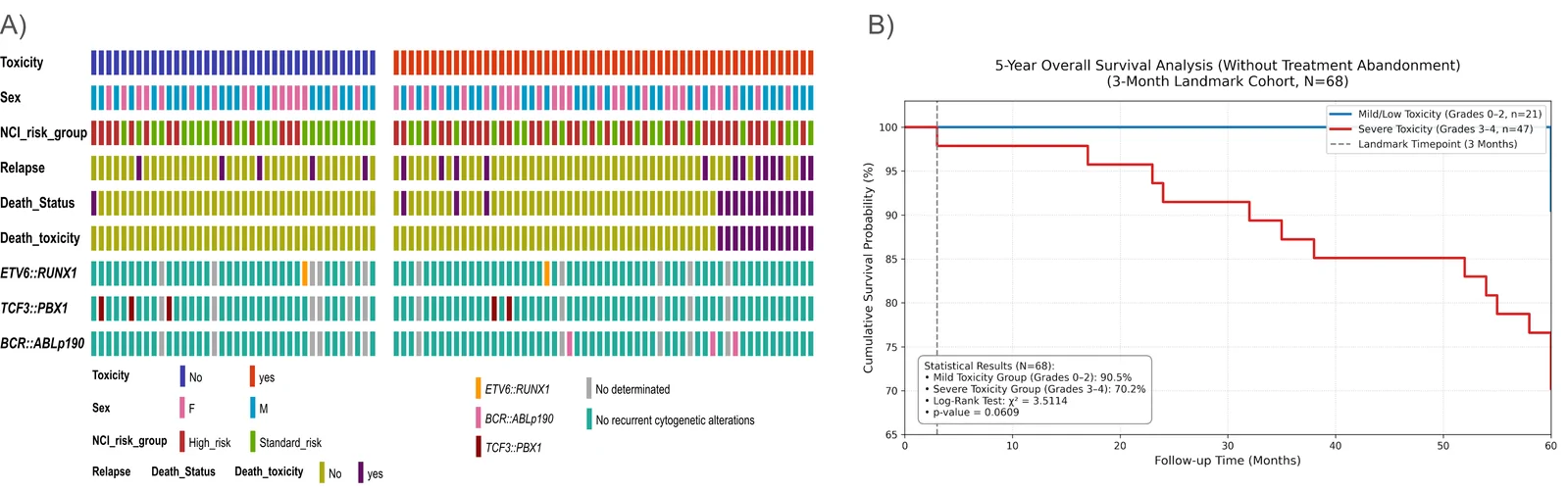
(**A**) Clinical characteristics of 96 pediatric patients with acute lymphoblastic leukemia (ALL) stratified by hematologic toxicity severity. Diagram showing clinical sex distribution, NCI risk classification (standard/high), recurrent cytogenetic alterations (*ETV6::RUNX1*, *TCF3::PBX1*, *BCR::ABL1 p^190^*), patients’ 5-year outcomes (alive, relapse, death, toxicity-related death), and hematologic toxicity severe (Grades 3–4), 64%, and mild (Grades 0–2), 36%, per CTCAE v5.0 criteria. Each column represents an ALL sample, and each characteristic or genetic alteration has its own row. (**B**) Landmark survival analysis at 100 days. Kaplan–Meier curves showing overall survival from the 100-day (3-month) landmark. One patient who died before the 100-day landmark was excluded. An additional 27 patients were right-censored at their last verified clinical contact before completing 60 months of follow-up. The log-rank test was used to compare survival distributions between groups (χ^2^ = 3.5114, *p*-value = 0.0609). Time is shown in months.

Of this cohort, 17.7% died during follow-up; among these deaths, a total of 15 deaths (15.9%) were documented in the severe hematologic toxicity group. Of these, 12 (80%) were classified as treatment-related mortality (TRM). No TRM events were recorded in the mild toxicity group. The median time from diagnosis to death was 35 months (range: 17–58 months), with 11 of 12 deaths (91.7%) occurring during maintenance therapy and one during remission induction. The immediate cause of death was septic shock in all 12 cases, with documented infection present in every patient; however, no specific pathogen was identified. All patients had Grade 4 neutropenia at the time of death. Two patients also presented with pneumonia, and one patient had neutropenic colitis. All 12 TRM events were adjudicated as ‘Definitely Related’ to chemotherapy according to NCI-CTCAE v5.0 criteria by two independent reviewers. The remaining 3 deaths (20%) were attributed to disease progression and were considered unrelated to treatment. Importantly, two patients who died from disease progression belonged to the mild toxicity group; therefore, 17 (17.7%) deaths were documented in the entire cohort. (Figure 1A and Supplementary Table S2).

A landmark survival analysis at 100 days was performed in 68 patients who survived the initial induction/consolidation phase. Of these, 21 patients (30.9%) experienced mild/low hematological toxicity (Grades 0–2), and 47 patients (69.1%) experienced severe toxicity (Grades 3–4). One patient who died before the 100-day landmark was excluded. Survival at 60 months was 90.5% in the mild toxicity group and 70.2% in the severe toxicity group, with no statistically significant difference between the groups (log-rank test, *p* > 0.05). Survival curves showed a parallel decline over time in both groups, indicating that the primary driver of mortality was disease progression rather than toxicity severity. A total of 27 patients (28.1%) were right-censored at their last verified clinical contact before completing 60 months of follow-up (Figure 1B). The primary reason for censoring was treatment abandonment during the maintenance phase, which occurred in patients from both toxicity groups: 13 of 34 patients (38.2%) in the mild toxicity group and 14 of 61 patients (23.0%) in the severe toxicity group abandoned treatment. The median time to abandonment was 44 months (range: 32–59 months).

### 2.3. ALL Patients Harbor More 3′ UTR Variants than Coding Region Variants

We performed targeted sequencing of exonic and 5′ and 3′ UTRs regions of 17 genes, and variant annotation and filtering were carried out across 96 pediatric patients with ALL. Sequencing quality control was assessed across 96 samples at both alignment and variant-calling levels. The cohort achieved a median target depth > 360× (IQR: 290×–430×), with >98.8% of targeted bases covered at ≥20× and >97.5% at ≥30×. Genotyping completeness was high, with an overall call rate > 96.0% (missingness < 4.0%), and allele balance for heterozygous calls showed a symmetric distribution centered at 0.48, with >97.1% within the 0.20–0.80 high-confidence window. Stringent per-genotype filters (GQ ≥ 20, DP ≥ 10×) retained >99.8% of calls, and site-level filtering followed GATK best practices. Comprehensive QC metrics are detailed in Supplementary Figure S3. All identified variants were annotated using Ensembl VEP, and variants were classified according to their genomic location (exonic, 5′ UTR, 3′ UTRs, intronic, and splice region) and predicted functional consequence; allele frequencies and genotype distributions across toxicity groups are provided. A complete summary of all identified variants across toxicity groups is provided in Supplementary Tables S3 and S4.

**Figure 2:**
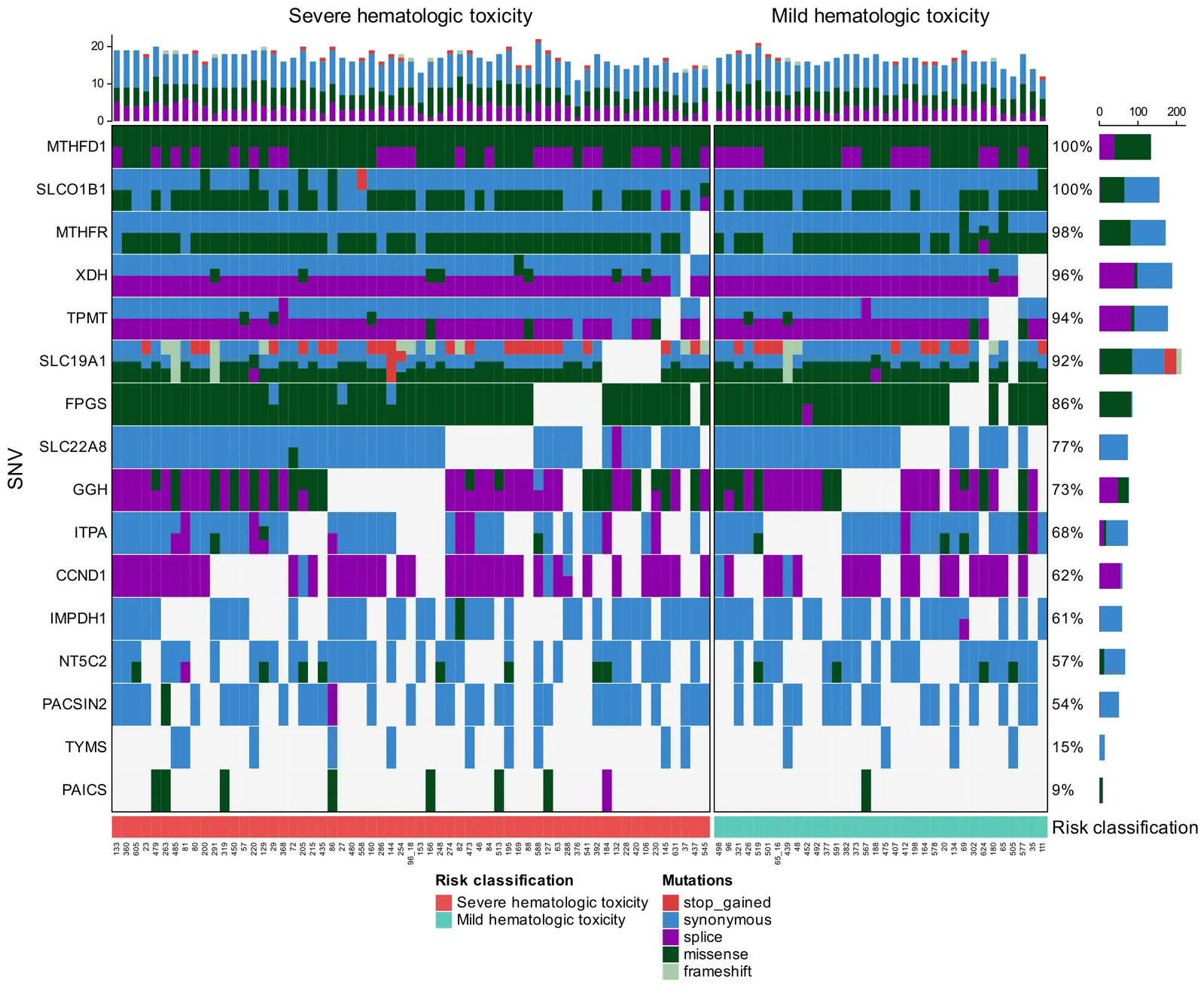
Distribution of variants in the exonic region across 17 pharmacogenes in a cohort of pediatric acute lymphoblastic leukemia (ALL) patients. The graph/table shows the percentage of patients carrying at least one variant in each of the 17 genes analyzed. The genes are ordered by decreasing variant frequency. Each column represents one patient, and the bottom bar plot indicates variant types.

**Figure 3:**
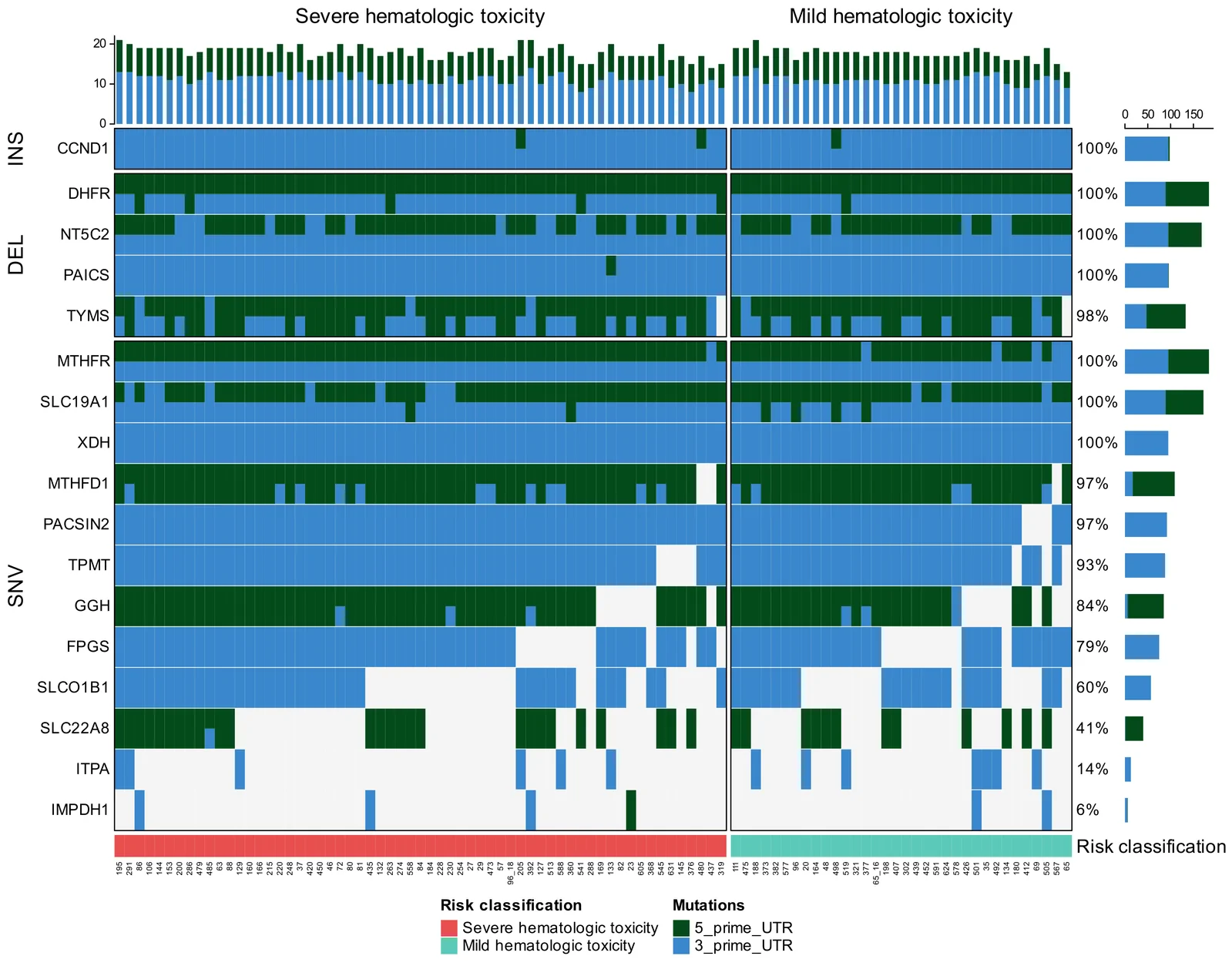
Distribution of variants in the 3′ and 5′ UTRs regions across 17 pharmacogenes in a cohort of pediatric acute lymphoblastic leukemia (ALL) patients. The graph/table shows the percentage of patients carrying at least one variant in each of the 17 genes analyzed. The genes are ordered by decreasing variant frequency. Each column represents one patient. SNV: single-nucleotide variant; DEL: deletion; INS: insertion.

A notable enrichment of variants was observed in 5′ and 3′ untranslated regions (5′ and 3′ UTRs). The genotyping analysis yielded a total of 8187 genotype calls across the cohort of 96 patients. This number represents the total number of calls and not the number of unique polymorphic loci, as a single variant may be observed in multiple individuals. The number of unique loci identified in this study was 439 (Supplementary Table S3). We found 452 (5.6%) indels and 7735 SNVs (94.4%). The majority (6059) of the genotype calls were located in 5′ and 3′ UTRs, compared to 2128 in coding regions. In the coding regions, there were 2098 SNVs and 30 indels. Within the coding regions, the 2128 genotype calls were distributed as follows: 13 (0.6%) frameshift, 684 (32.1%) missense, 359 (16.8%) splice, 33 (1.5%) stop-gained, and 1039 (48.8%) synonymous (Figure 2). Overall, 4900 genotype calls were located in the 3′ UTR region, and 1159 were located in the 5′ UTR region. Specifically, 5′ and 3′ UTRs harbored 5637 SNVs and 422 indels (Figure 3). The analysis of normalization for callable length showed that the 3′ UTR region showed a variant density of 14.6 variants/kb, and the 5′ UTR region had a density of 13.4 variants/kb, compared to 3.2 variants/kb in the coding regions. This represents a 4.1–4.5-fold difference, indicating enrichment in the 3′ and 5′ UTRs (Supplementary Table S5). The most frequently altered gene was *SLC19A1*, followed by the *MTHFR*, *DHFR*, *XDH*, *NT5C2*, *PACSIN2*, *TPMT*, *MTHFD1*, *SLCO1B1*, *CCND1*, *PAICS*, *TYMS*, *GGH*, *FPGS*, *ITPA*, *SLC22A8*, and *IMPDH1* genes. Variants were classified as pathogenic or likely pathogenic according to ACMG/AMP criteria and were predicted to be damaging by at least three in silico tools (SIFT, PolyPhen-2, and REVEL), with *TPMT*, *PAICS*, and *SLC19A1* harboring exclusive deleterious variants. *SLC19A1* showed both the highest frequency and high pathogenicity burden, including a stop-gained variant (rs114832456) and multiple deleterious missense mutations. The *TPMT*, *PACSIN2*, *IMPDH1*, *GGH*, *PAICS*, *SLC22A8*, *NT5C2*, *CCND1*, *TYMS*, and *FPGS* genes show alterations exclusively in ALL patients who have severe hematologic toxicity. In contrast, the *DHFR* gene showed an opposite profile, with 15 variants in 5′ and 3′ UTRs regions exclusively in patients without severe toxicity, suggesting a potential protective effect.

### 2.4. Variants in Genetics Associated with Severe Hematologic Toxicity

A total of 96 patients (34 with mild toxicity and 62 with severe hematologic toxicity) were included in the genetic association analyses for 17 pharmacogenes. All variants were in Hardy–Weinberg equilibrium when analyzed within the full cohort. However, given the limited sample size and the stratification by phenotype group, these HWE results were not interpreted as standalone evidence of genotyping accuracy. They were considered alongside other QC parameters (call rates, allele frequencies, and cluster plot inspection), all of which collectively supported the overall quality of the genotype calls. A complete summary of our multivariate regression analysis, including genotype distributions and full association statistics (OR, 95% CI, and *p*-values for all genetic models) across all 17 genes, is provided in Supplementary Table S4. Among the 17 genes analyzed, we observed the strongest nominal associations with severe hematologic toxicity for variants in *TYMS* (rs45445694) (overdominant model: OR = 3.21; 95% CI = 1.23–8.38; *p* = 0.014 and codominant model: OR = 3.31; 95% CI = 1.17–9.34; *p* = 0.047) and *CCND1 LD block* (rs678653/rs7177) (overdominant model: OR = 2.60; 95% CI = 1.01–6.71; *p* = 0.044). Importantly, these associations remained nominally significant after adjusting for treatment protocol, sex, age, rearrangements, and WBC count per mm^3^, indicating that the genetic effects were not entirely driven by interinstitutional differences in clinical management or characteristic clinical features of patients. Therefore, these genes were selected for detailed discussion also due to their established biological relevance in MTX pharmacology and previous associations with chemotherapy toxicity in pediatric ALL [17,18,19]. Although the variants rs45445694 (*TYMS*) and the *CCND1* LD block (rs678653/rs7177) showed associations with severe hematologic toxicity (*p* < 0.05), none remained statistically significant after rigorous correction for multiple testing. A comprehensive Bonferroni adjustment across 17 genes and 5 genetic models yielded a corrected significance threshold of α = 0.000588, and neither variant reached this threshold. Therefore, these findings should be considered exploratory and hypothesis-generating, requiring independent replication in larger cohorts before any clinical conclusions can be drawn.

**Figure 4:**
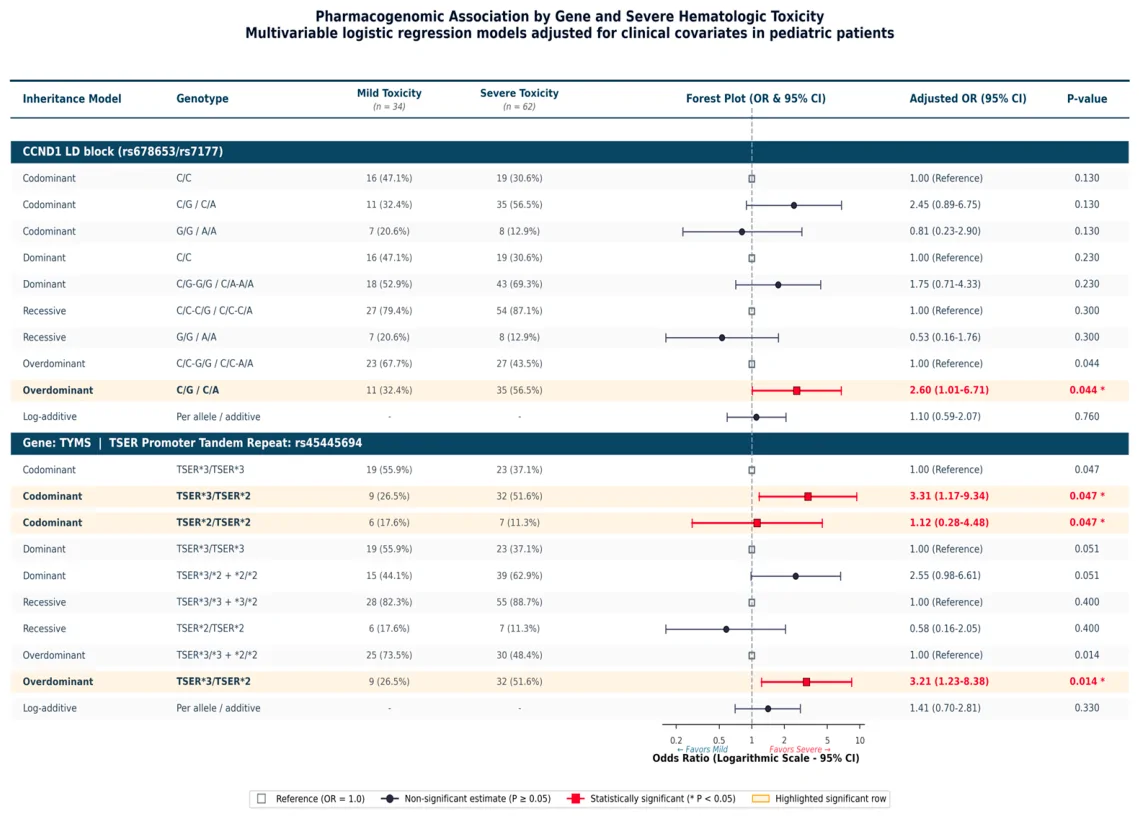
Forest plot of pharmacogenomic nominal associations with severe hematologic toxicity under multiple inheritance models. Multivariable logistic regression analysis was adjusted for clinical covariates in 96 pediatric patients. The plot displays odds ratios (ORs) with 95% confidence intervals (CIs) for variants in *CCND1* linkage disequilibrium block (rs678653/rs7177) and *TYMS* TSER promoter enhancer tandem repeat polymorphism (rs45445694). Five inheritance models were evaluated for each variant: codominant, dominant, recessive, overdominant, and log-additive (additive per allele). The red dot indicates statistically significant findings (*p* < 0.05). The yellow-highlighted rows showed the strongest associations. Genotype frequencies are presented as counts and percentages for each toxicity group (mild, *n* = 34; severe, *n* = 62).

The group of patients with severe toxicity had a significantly higher percentage of rs45445694 in the *TYMS* gene and *CCND1* LD block (rs678653/rs7177) than those with mild hematologic toxicity. The association analysis forest chart of *CCND1* and *TYMS* genes is presented in Figure 4.

In the *TYMS* gene, rs45445694 was nominally significantly associated with hematologic toxicity, showing a higher percentage of heterozygous TSER*3/TSER*2 genotype in patients with severe hematologic toxicity (51.6%) compared to mild hematologic toxicity (26.5%). Under the overdominant model, heterozygous carriers (TSER*3/TSER*2) exhibited a 3.31-fold increased risk of severe hematologic toxicity compared to homozygous reference individuals (TSER*3/TSER*3) (OR = 3.31, 95% CI: 1.17–9.34, *p* = 0.047), whereas homozygous variant carriers (TSER*2/TSER*2) did not show a significant risk increase. This pattern was further supported by the overdominant model, which directly compares heterozygotes against both homozygotes combined, yielding a 3.21-fold increased risk for heterozygous carriers (OR = 3.21, 95% CI: 1.23–8.38, *p* = 0.014). Collectively, these results indicate a specific heterozygous effect, where carrying one copy of the variant allele confers a higher risk of severe hematologic toxicity than carrying either zero or two copies (Figure 4). In the *CCND1* gene, rs678653 and rs7177 exhibited nominally significant associations with severe hematologic toxicity. Linkage disequilibrium analysis revealed that these were found to be in near linkage disequilibrium (D′ = 0.9997, r^2^ = 0.9997), indicating that they represent essentially the same genetic signal. Under the overdominant model, heterozygous carriers (0/1) of these variants showed a 2.60-fold increased risk of severe toxicity compared to homozygous individuals (OR = 2.60, 95% CI: 1.01–6.71, *p* = 0.044). The codominant model for these variants did not reach statistical significance (*p* = 0.13), suggesting that the heterozygous effect is the predominant genetic model for this locus (Figure 4 and Supplementary Table S4).

Collectively, the analyses identified promising but exploratory associations for variants in the cell cycle regulator *CCND1* (rs678653/rs7177) and the folate metabolism gene *TYMS* (rs45445694) with severe hematologic toxicity in this Mexican pediatric ALL cohort. However, given the relatively limited sample size (*n* = 96) and the lack of formal correction for multiple testing, these findings should be interpreted as hypothesis-generating and require validation in a larger cohort. The genotypes for rs678653 in *CCND1* (Supplementary Figure S1) and the 3R allele in *TYMS* (Supplementary Figure S2) were confirmed by Sanger sequencing using the primers described in Supplementary Table S6.

### 2.5. Variants by Gene TYMS and CCND1 Genes

**Figure 5:**
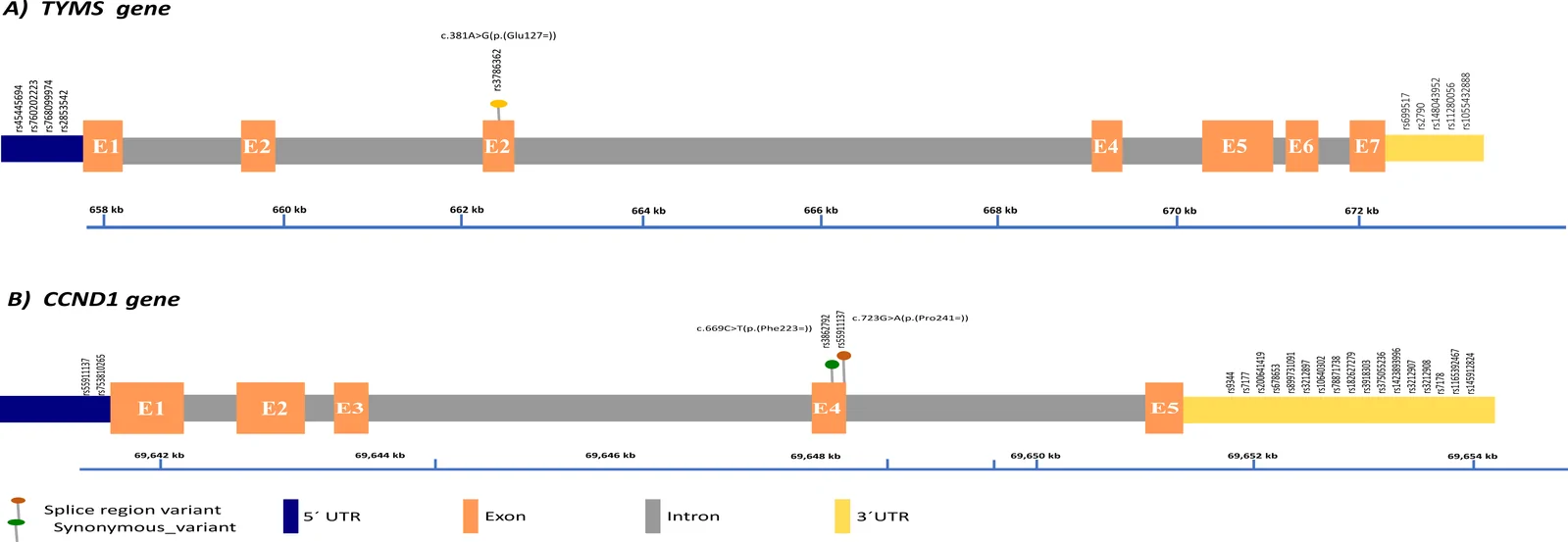
*CCND1* and *TYMS* variant spectrum. The schematic diagram summarizes the genetic abnormalities in (**A**) the *TYMS* gene and (**B**) *CCND1* and their locations on the chromosome that were detected in 96 patients with ALL. The numbered exons and orange color correspond to coding regions, and those with a yellow color correspond to the 3′ and 5′untranslated regions. E = Exon.

In this study, all gene exons and their 3′ and 5′ UTRs regions were sequenced for 17 pharmacogenes of 96 pediatric patients with ALL. The data analysis showed variants principally in the 5′ and 3′ UTRs regions, followed by variants, in smaller numbers, in exonic regions in all genes. A total of 11 gene variants in the *TYMS* gene were found; of these, 4 were in 5′ UTR, 5 were in 3′ UTR, and only 1 synonymous variant was observed (Figure 5A). For the *CCND1* gene, 2 were in 5′ UTR, 17 were in 3′ UTR, 1 was a synonymous variant, and 1 was related to alternative splicing (Figure 5B).

## 3. Discussion

Acute lymphoblastic leukemia (ALL) is the most common cancer in pediatric populations, and treatment response has been related to interindividual differences in susceptibility to adverse toxic effects, which are mainly associated with pharmacogenetic variation. The avoidance of severe toxicity is a critical determinant of successful treatment in ALL patients, and the identification of genomic variants constitutes an important tool for identifying phenotypes associated with adverse effects, such as toxicity [20]. In this regard, only two Mexican studies have specifically reported this outcome, but with minor percentages. Martín-Trejo et al. reported that 7.1% of all deaths were directly attributable to chemotherapy toxicity, and in a national analysis, a 5.3% induction mortality was reported, which encompasses deaths from toxicity, infections, and bleeding complications during the initial treatment phase. A TRM rate of 12.5% is notably higher than the 2–5% TRM reported in contemporary pediatric leukemia trials, suggesting that our cohort may have been enriched for high-risk patients or that toxicity was more severe than anticipated. All TRM events were attributable to septic shock in the context of profound, prolonged neutropenia, underscoring the critical importance of infection prevention and early intervention in patients with severe cytopenias. The fact that 91.7% of TRM events occurred during maintenance therapy, when patients are typically considered to be at lower risk, is particularly striking and warrants further investigation. It is also noteworthy that no specific pathogen was identified in any case, which may reflect the limitations of routine microbiological diagnostics or the use of broad-spectrum empirical antibiotics. These findings highlight the need for improved strategies for infection surveillance and supportive care in patients with severe hematological toxicity, particularly during the maintenance phase of treatment. Furthermore, the two deaths attributed to disease progression occurred in patients with mild toxicity, reinforcing that mortality in this group was driven by the underlying malignancy rather than treatment-related complications. All 12 treatment-related deaths occurred in the severe toxicity group, primarily due to sepsis during neutropenia (75%) and intracranial hemorrhage from thrombocytopenia (25%), underscoring the critical need for improved prevention strategies. The analysis showed the occurrence of Grade 3/4 neutropenia in 100% of severe patients and high rates of Grade 4 thrombocytopenia (61.3%) and anemia Grade 3/4 (79.0%), demonstrating that myelosuppression is not a self-limited event but a recurrent and persistent challenge. Supportive care utilization was intensive, with 88.7% requiring transfusions and 45.2% receiving Filgrastim, reflecting both the severity of toxicity and the substantial economic burden on the healthcare system. Notably, in the “mild toxicity” group, 35.3% had Grade 3/4 neutropenia, and 29.4% required transfusions, emphasizing the need for lineage-specific and granular toxicity reporting. These findings align with previous Mexican studies reporting high induction mortality and lower survival rates, providing a mechanistic explanation for survival disparities through toxicity-related treatment delays and dose reductions. Additionally, toxicity occurs in both early (induction/consolidation) and maintenance phases. This demonstrates that toxicity is not a single event but a recurrent phenomenon. Neutropenia is the most frequent event in both phases. This reinforces the relevance of the selected genes, as MTX and 6-MP are well-known causes of neutropenia. Thrombocytopenia and anemia are more frequent in the early phase. This is expected, as drugs such as anthracyclines and L-asparaginase used during induction affect these lineages more profoundly.

In the landmark survival analysis, severe hematological toxicity during the first 100 days of treatment was not associated with inferior overall survival. This encouraging finding suggests that, with appropriate supportive care—including transfusions, growth factors, and infection management—patients who experience severe cytopenias can achieve excellent long-term outcomes. The high rate of treatment abandonment (27 patients, 39.7%) represents a significant limitation of the analysis and reflects the challenges of treating a pediatric population in a resource-limited setting. Nevertheless, these findings underscore the importance of aggressive supportive care during early treatment phases and highlight that severe hematological toxicity should not be viewed as a barrier to achieving long-term survival, provided that patients receive adequate supportive measures.

This study reveals exploratory nominal associations between germline genetic variants and severe hematologic toxicity that require validation in independent cohorts. Germline variants are inherited and often confer high-penetrance susceptibility to drug-related toxicities, commonly affecting key cellular processes such as replication, apoptosis, cell cycle regulation, cytoskeletal dynamics, nucleic acid metabolism, and signal transduction [21]. Among the various types of genomic variation (single-nucleotide variants (SNVs), insertions, deletions, and copy number variations), SNVs are the most frequent, accounting for approximately 90% of all human genetic variation. An SNP is a single-nucleotide change present in at least 1% of the population, and although over 12 million SNPs are estimated to exist, only ~60,000 are located within coding regions [22]. This highlights the relevance of systematically evaluating both coding and non-coding genomic variants to better predict severe toxicity and personalize ALL therapy.

The present study sought to evaluate whether genetic variation in genes involved in methotrexate (MTX) and 6-mercaptopurine (6-MP) metabolism contributes to susceptibility to severe hematologic toxicity in the Mexican population. The important finding is the outstanding enrichment of variants in 5′ and 3′ untranslated regions (5′ and 3′ UTRs) compared to coding sequences (CDSs), consistent with the well-established principle that coding regions are under stronger purifying selection than non-coding regions. Regulatory regions are expected to tolerate a higher burden of genetic variation, elucidating the observed higher variant density in these regions [16]. The 5′ and 3′ UTRs regions are critical determinants of post-transcriptional regulation—the 5′ UTR controls translation initiation, while the 3′ UTR harbors binding sites for microRNAs and regulatory elements that modulate mRNA stability and subcellular localization [23,24]. Variants in 5′ and 3′ UTRs regions can subtly alter protein expression levels, with pharmacologically relevant consequences for drug efficacy and toxicity, and variants in these regions have been associated with diverse human diseases [25]. The identification of rs45445694 in the *TYMS* enhancer region is particularly noteworthy, given the extensive experimental evidence linking TSER genotype to *TYMS* expression and TS enzyme activity. This supports the biological plausibility of our observed association with hematologic toxicity. In contrast, the *CCND1* 3′ UTR variants identified in our study remain functionally uncharacterized, and their potential role in regulating *CCND1* expression or miRNA interactions requires experimental validation. These variants should therefore be considered candidate markers warranting further investigation.

Most genomic association studies to date have prioritized exonic variants, leaving the contribution of 5′ and 3′ UTRs variants to chemotherapy-induced toxicity largely underexplored. Notably, a recent large-scale functional study in pediatric ALL revealed that over 500 non-coding variants exhibit allele-specific regulatory activity and identified 54 variants with high-confidence connectivity to target genes via chromatin looping. Several of these variants were functionally validated to modulate sensitivity to antileukemic agents such as vincristine [26]. These findings underscore that the non-coding genome, including 5′ and 3′ UTRs, harbors a substantial and previously underappreciated source of pharmacogenetic variation relevant to treatment outcomes and toxicity in childhood ALL. Our data demonstrate that this regulatory landscape constitutes a substantial reservoir of genetic variability that remains obscure when studies focus exclusively on exonic or common VSN variants. We identified several variants in the 5′ and 3′ UTRs of *CCND1* and *TYMS* genes that showed nominal associations with severe hematologic toxicity in a Mexican pediatric cohort of patients with acute lymphoblastic leukemia (ALL). For *CCND1*, the variants rs678653 and rs7177 are in nearly complete linkage disequilibrium and were associated with severe toxicity under the overdominant model, suggesting that heterozygous carriers might be at increased risk of severe toxicity in hematology. This correlated with the known role of cyclin D1, encoded by *CCND1*, as a critical modulator of the cell cycle to allow for the progression from the G0 phase to the synthesis S phase [27]; therefore, perturbations in this pathway could modulate the susceptibility of hematopoietic progenitors to chemotherapy-induced damage.

In the cyclin D1 gene (*CCND1*), variants have been extensively implicated in cancer susceptibility and treatment outcomes across multiple tumor types. For instance, rs678653 and rs9344 (G870A) were significantly associated with susceptibility to childhood acute lymphoblastic leukemia (ALL) in a Taiwanese pediatric cohort, with the AG and GG genotypes conferring a protective effect compared to the AA genotype [28]. In esophageal squamous cell carcinoma, the GA+AA genotype of G870A increased the risk of developing the disease by nearly 3-fold (OR = 2.80), and the CC genotype of the G1722C variant (rs678653) was also associated with increased susceptibility (OR = 2.58) [29]. The heterozygous genotype of rs7177 was associated with a 1.17-fold increased risk of colorectal cancer, while the G allele of rs9344 conferred a 1.24-fold higher risk of renal cell carcinoma in the Chinese population [30]. In breast cancer, rs7177 is one of seven variants associated with histological Grade 3 (high-grade malignancy), with an OR of 1.5 [31]. Particularly, the G870A variant in pediatric ALL patients receiving high-dose methotrexate was found to have heterozygous carriers (AG) who had a 6.39-fold increased risk of hepatotoxicity, while the combined AG/AA group showed a 4.44-fold increased risk compared to GG homozygotes [17]. However, in our Mexican cohort, the G870A variant was not associated with severe hematologic toxicity in our study. Several factors may explain this inconsistency: we did not evaluate hepatotoxicity; our relatively small sample size reduced the statistical power to detect variants with low allele frequencies or small effect sizes; and the inclusion of patients from different ethnic backgrounds, along with environmental or dietary factors, may have influenced methotrexate metabolism. These findings and our exploratory results in this cohort add to the growing evidence that *CCND1* variability may play an important role in modulating cancer susceptibility and chemotherapy-associated adverse effects.

In relation to the *TYMS* gene, this gene encodes the enzyme thymidylate synthase (TS), a key enzyme in DNA synthesis and an important target for chemotherapy drugs such as 5-fluorouracil and methotrexate (MTX) [18]. MTX inhibits de novo purine synthesis and acts as a prodrug that requires intracellular polyglutamylation (MTXPGs) to achieve its cytotoxic effects. Once converted to MTXPGs, they cause greater inhibition of target enzymes, including TS [32]. It has been demonstrated that the intracellular accumulation of methotrexate polyglutamates correlates with prolonged inhibition of thymidylate synthesis in vitro [33]. Thus, the *TYMS* gene plays a critical role in the pharmacogenomics of MTX in patients with ALL. Genetic variants in the *TYMS* gene that affect the enzyme’s structure, activity, or expression can influence leukemic cell sensitivity or resistance to MTX, thereby impacting both treatment efficacy and the risk of severe toxicity in pediatric patients. In the present study, the variant rs45445694, with alleles 2R and 3R, was associated with a 3.21-fold increased risk of severe hematologic toxicity in heterozygous carriers. It is located in the *TYMS* promoter enhancer region and is a variable number tandem repeat (VNTR) of 28 base-pair repeats known as TSER, also known in the literature as rs34743033. The most common alleles are TSER*2 (2 R) and TSER*3 (3 R), and they have been directly related to the transcriptional regulation of *TYMS*; the 3R allele confers higher translational efficiency and, consequently, higher TS enzyme levels compared to the 2R allele [19]. Our exploratory findings show some overlap with previous reports. In a cohort study of 64 Jordanian children with ALL, the authors reported a significant association between thrombocytopenia and 3R/3R homozygotes (rs45445694). This showed a 3.3-fold increased risk compared to 2R carriers [34]. Although the specific hematological outcome differed (thrombocytopenia vs. severe hematologic toxicity), both studies identified rs45445694 as a modulator of MTX-induced myelosuppression. This convergence highlights the importance of *TYMS* regulatory variants in modulating hematopoietic toxicity. In contrast, Oosterom et al. studied the same rs45445694 (rs34743033/TSER) in a cohort of 108 Dutch children with ALL and found no significant association with MTX-induced oral mucositis. The discrepancy may be explained by differences in the phenotypic (hematologic toxicity vs. oral mucositis) and ethnic composition of the cohorts [35].

Beyond toxicity, *TYMS* variation has been associated with outcomes in ALL. In a study of 205 French Canadian children, it was demonstrated that homozygosity for the 3R allele (*TSER3/TSER3*) was associated with a risk of relapse or death and shorter event-free survival compared to patients with other genotypes [36]. Likewise, a prospective multicenter study of 354 Polish children with ALL found a significant association between the same rs45445694, with *TSER3/TSER3* patients showing a higher relapse rate [37]. These results indicate that high-expression *TYMS* genotypes (*TSER3/TSER3*) may confer a poorer prognosis, probably by promoting leukemic cell survival and resistance to MTX, because higher TS enzyme levels require greater MTX concentrations to achieve adequate inhibition. The consistent association of rs45445694 with prognosis in different cohorts emphasizes the biological and clinical relevance of this polymorphism. Additionally, the *TYMS* 3′ UTR 6 bp insertion/deletion polymorphism (rs151264360) also contributes to the pharmacogenomic landscape. Kealey et al. demonstrated that in northwestern European adults, del/del homozygotes exhibited higher RBC folate and lower homocysteine levels compared to ins/ins or ins/del carriers. suggesting that *TYMS* variation may influence folate homeostasis and, indirectly, MTX-related toxicity [38]. Kotur et al. showed that pediatric ALL with *TYMS* 6bp deletion was more likely to experience gastrointestinal toxicity, considering it as an important pharmacogenomic marker of MTX response, further reinforcing the clinical relevance of *TYMS* genetic profiling in ALL patients [39]. The clinical relevance of *TYMS* genetic variation is not limited to ALL. Studies in other malignancies and therapies reinforce the notion that *TYMS* genetic profiling may have clinical utility in different cancer types, and the specific variants and their effects may vary depending on the drug, tumor type, and population. Saif et al. demonstrated that *TYMS* low expression genotypes and the 3′ UTR del/del genotype were significantly associated with severe (Grade 3–4) adverse events, including diarrhea, mucositis, and myelosuppression, in a cohort of 430 patients with solid tumors treated with 5-fluorouracil or capecitabine [40]. Lin et al. reported that the CT genotype of *TYMS* rs2853741 increased the risk of capecitabine-induced hand–foot syndrome in 342 Chinese patients with metastatic breast cancer [41]. In colorectal cancer, Martinez-Balibrea et al. identified the *TYMS* 3R/3R genotype as an independent predictor of tumor response in patients treated with irinotecan and 5-FU combination therapy (OR = 5.87, *p* = 0.005) [42].

One of this study’s strengths is that children with ALL were consecutively enrolled from different hospitals in Mexico City; therefore, the distribution of genes and frequency of toxicity mainly reflect these phenomena in our pediatric population. This allows us to extrapolate these results to other Latin American populations with these same characteristics. Another pharmacogenomic study conducted in the same Mexican pediatric B-ALL cohort also identified genetic variants associated with hematologic adverse events, specifically in membrane transporter genes of the ABC and SLC families. The results indicated that variants in ABCC1 (rs129081), ABCC4 (rs2274409), and ABCC5 (rs939338, rs1132776, rs3749442, rs4148575, rs4148579, and rs4148580) were associated with an increased risk of severe hematologic toxicity [43]. These results complement our study and demonstrate the complex etiology of chemotherapy-related toxicity in the Mexican population. This result is initial corroboration for developing a polygenic model of severe toxicity risk in Mexican children with ALL, where the cumulative effect of multiple genetic variants, each one with its individual effect, may determine the overall susceptibility. The principal objective of pharmacogenetics is to develop polygenic risk scores (PRSs) that accurately predict drug response and toxicity for individual patients, enabling prospective personalization of treatment regimens to enhance efficacy and safety [44]. Although PRS models have been successfully implemented for diseases such as obesity and schizophrenia, their utility in patients with ALL remains under-researched [45,46,47,48]. Moreover, the transferability of PRS models of non-Latino populations to Latino ones has been shown to be deficient, emphasizing the need for ancestry-specific models [44].

Although Grade 3–4 myelosuppression is an expected consequence of intensive chemotherapy regimens used in pediatric ALL, it is related to the obligatory bone marrow suppression required to achieve leukemic remission [5,6]. In our cohort, patients who developed severe hematologic toxicity experienced significantly higher rates of neutropenia (any G3/G4 = 100% vs. 35.3%); thrombocytopenia (any G3/G4 = 93.5% vs. 41.2%); anemia (any G3/G4 = 79% vs. 35.3%); transfusion requirements (88.7% vs. 29.4%); and overall mortality (12.5%) compared to those with mild hematologic toxicity. Therefore, the ability to prospectively identify those at high risk of developing severe toxicity would enable personalized supportive care strategies. The value of genetic biomarkers, including the *TYMS* and *CCND1* variants identified in this study, could potentially help to stratify patients beyond the predictable myelosuppression of chemotherapy, if validated in future studies. Specifically, they could help to identify individuals with germline variants that predispose them to more severe hematologic toxicity, thereby optimizing supportive care and planning dose modifications before complications arise. Although these associations are exploratory and require validation, they represent a step toward precision medicine to identify patients who may benefit from preemptive intervention, particularly in resource-limited settings, where the consequences of severe myelosuppression are most pronounced. We suggest that *CCND1* and *TYMS* represent candidate pharmacogenetic markers worthy of further investigation in larger cohorts and well-powered studies, given their exploratory associations in this cohort.

### Limitations

We acknowledge the following as limitations of our study: We lack data on baseline renal function, including serum creatinine, estimated glomerular filtration rate (eGFR), and plasma methotrexate (MTX) levels at 24, 48, or 72 h post-infusion, which were not uniformly collected across the participating hospitals, representing a limitation of our study. Likewise, we consider that future prospective studies should incorporate standardized pharmacokinetic monitoring and baseline renal function assessment to resolve the interplay between genetic variation, renal clearance, and toxicity. Furthermore, the small sample size (*n* = 96) resulted in limited numbers of patients in certain genotype categories, particularly for homozygous variant carriers (*TYMS* *TSER2/TSER2*: *n* = 9; *CCND1* 1/1: *n* = 11). Consequently, the confidence intervals for odds ratios are wide, and our study is underpowered to detect modest effect sizes. Therefore, these findings remain exploratory and require validation in larger cohorts.

## 4. Materials and Methods

### 4.1. Patients

This study included peripheral blood from 96 patients diagnosed with ALL in public hospitals in Mexico City who were treated with the standard Berlin–Frankfurt–Munich (BFM) or St. Jude Total Therapy XIII-B HR protocols. The patients were categorized into two groups: patients with severe hematologic toxicity (cases) and mild-grade hematologic toxicity (controls). Hematologic toxicity was graded according to the Common Terminology Criteria for Adverse Events (CTCAE) v5.0. We defined severe hematologic toxicity as CTCAE Grade 3–4, consistent with international standards and clinically validated by the poor outcomes observed in patients meeting this criterion in our cohort. The CTCAE defines Grade 3 as severe or medically significant but not immediately life-threatening; hospitalization or prolongation of hospitalization is indicated, along with disabling and limiting self-care Activities of Daily Living (ADLs). Grade 4 represents life-threatening conditions demanding urgent intervention. Although Grade 3–4 myelosuppression is a recognized consequence of intensive chemotherapy, in Mexican patients, these events carry particular clinical significance. In our Mexican cohorts, where treatment-related mortality is 7.1% and induction mortality is 5.3%, even Grade 3–4 events have severe consequences due to limited access to intensive supportive care, higher rates of malnutrition, and increased susceptibility to infectious complications. All deaths were adjudicated by two independent clinical oncologists who were blinded to the genotyping results. Adjudication was based on a review of all available clinical records, including laboratory values and clinical notes. Discrepancies between reviewers were resolved by consensus discussion with a third reviewer. All 12 treatment-related deaths were classified as ‘definitely related’ to chemotherapy. The severe hematological toxicity cohort consisted of 62 patients, each having at least one sign of Grade 3–4 toxicity. The control cohort included 34 patients with Grade 0–2 toxicity.

### 4.2. Landmark Survival Analysis

We conducted the landmark analysis as follows: Redefinition of Exposure Group (100-Day Landmark): Toxicity severity was defined strictly based on adverse events occurring within the first 100 days of treatment (induction/consolidation); the majority occurred during the maintenance phase (ranging from Month 17 to Month 58 of treatment); only one case of death occurred immediately after induction. For patients surviving the initial 100-day evaluation period, longitudinal overall survival was analyzed onward, considering disease-related mortality (leukemia relapse/progression) as the primary endpoint. Additionally, of the 95 patients enrolled, 69 contributed to the five-year survival analysis. The remaining 27 patients were right-censored at their last verified clinical contact before completing 60 months of follow-up. Censoring was predominantly due to treatment abandonment during the extended maintenance phase or loss to follow-up. Overall survival from the 3-month landmark onward was estimated using the Kaplan–Meier method and compared via the log-rank test.

### 4.3. Library Preparation, Targeted Capture, and Next-Generation Sequencing

Peripheral blood DNA was extracted using the QIAamp^®^ Blood Mini Kit (Qiagen, Hilden, Germany) according to the manufacturer’s protocol. Genomic DNA samples were quality-checked on TapeStation (Agilent Technologies, Santa Clara, CA, USA) and quantified using the Qubit dsDNA BR Assay (Thermo Fisher Scientific Inc., Waltham, MA, USA). A custom panel was designed using Agilent SureDesign software ver 5.0 (Agilent, Santa Clara, CA, USA), which covered the *GGH*, *PACSIN2*, *SLC19A1*, *DHFR*, *SLC22A8*, *PAICS*, *MTHFD1*, *CCND1*, *IMPDH1*, *NT5C2*, *FPGS*, *TPMT*, *ITPA*, *TYMS*, *MTHFR*, *XDH*, and *SLCO1B1* genes, and the final design covered 99% of the target region. These genes are involved in the metabolism of methotrexate and 6-mercaptopurine, in accordance with the evolving literature. The complete coding DNA sequence and the 5′ and 3′ UTRs were sequenced using an enrichment system based on capture with probes, SureSelect (Agilent) aired-end (PE) libraries were constructed with 100 ng of genomic DNA, and libraries were sequenced on an Illumina NextSeq500 instrument (Illumina, San Diego, CA, USA), generating 151 bp paired-end reads.

### 4.4. Sequencing Data Analysis

Analysis was performed using a bioinformatics pipeline. First, FASTQ files were checked for read quality using FastQC v0.12.1, and adapters and low-quality reads were trimmed using Trimmomatic. Afterward, high-quality paired-end reads were aligned to the reference human genome (build GRCh38) using the Burrows–Wheeler Aligner v0.7.17, and single-nucleotide variants and indels were identified using the Genome Analysis Toolkit v4.5. (GATK HaplotypeCaller). Filtering of SNPs and indels was performed using BCFtools v1.18, and the variants were annotated with SnpEff. Sequencing QC was performed at two levels: (1) alignment and coverage using mosdepth v0.3.4 on targeted pharmacogenomic regions and (2) variant and sample-level genotyping QC from the multi-sample VCF using bcftools v1.18. Regarding variant density analysis and length-based normalization, to enable a valid comparison of variant distribution across different genomic regions (5′ and 3′ UTRs, and coding sequences), we performed a length-based normalization, calculating the variant density as the number of unique variants per kilobase (kb) of callable sequence for each region: Variant density = (Number of unique variants in the region)/(Total callable length of the region in kb). All analyses were performed using R for genomic interval manipulation and annotation. All variants were annotated using the Ensemble Variant Effect Predictor (VEP; v110) with the following plugins: dbNSFP (for in silico predictions including SIFT, PolyPhen-2) and ClinVar (for clinical significance). Population frequencies were obtained from gnomAD v3.1. Variants were classified as Pathogenic, Likely Pathogenic, Variant of Uncertain Significance (VUS), Likely Benign, or Benign according to the 2015 ACMG/AMP guidelines.

### 4.5. Statistical Analyses

For each variant call that met quality and frequency criteria (MAF ≥ 1%, genotype call rate ≥ 90%, HWE *p* ≥ 0.05), association with severe hematologic toxicity was tested under five inheritance models using the online program SNPStats (https://www.snpstats.net/, http://bioinfo.iconcologia.net/SNPStats, accessed on 1 July 2023).

All patients received chemotherapy according to either the St. Jude Total XIII-B or BFM protocols, which present variations. To account for potential confounding by institutional differences, we included the treatment protocol (St. Jude vs. BFM) as a covariate in all multivariate regression models. For this, a correlation matrix was constructed using the following as covariates: sex, age, rearrangements, treatment protocol, and WBC count per mm^3^ at diagnosis. The SNPStats program allowed us to identify the strength of association by obtaining the odds ratio (OR) with a confidence interval, with statistical significance set at *p* < 0.05. All five inheritance models (codominant, dominant, recessive, overdominant, and log-additive) were tested for each genotype call. To control for type I error due to multiple comparisons, we applied Bonferroni correction across all variants and all inheritance models tested.

### 4.6. Validation of Mutations by PCR and Sanger Sequencing

The *TYMS* and *CCND1* variants identified by the NGS custom capture were validated. Specific primers were designed to amplify the region using Primer3Plus softwarev3.2 (Supplementary Table S6). We performed high-fidelity PCR (UCP HiFidelity) (Qiagen, Hilden, Germany), and the PCR products were cloned into the pJET1.2 vector using the CloneJET PCR Cloning Kit (Thermo Fisher Scientific, Waltham, MA, USA). The plasmids were confirmed by Sanger sequencing using an ABI3500xL genetic analyzer (Thermo Fisher Scientific).

## 5. Conclusions

In this Mexican pediatric ALL cohort, the application of sequencing methodologies improved the detection of pharmacogenes and revealed variants in 5′ and 3′ UTRs regions associated with the development of severe hematologic toxicity. Analysis strategies, which include NGS to improve detection and characterization and construct clinically relevant prediction models, increase the robustness of prediction of toxicity. The contribution of these variants to severe hematologic toxicity in ALL requires a cautious interpretation, aimed at validating these findings, identifying novel regulatory associations, and integrating multiple genes to construct clinically relevant prediction models.

## Data Availability

The original contributions presented in this study are included in the article/Supplementary Material. Further inquiries can be directed to the corresponding authors.

## Supplementary Materials

The following supporting information can be downloaded at: https://www.mdpi.com/article/10.3390/ijms27188131/s1.

## Abbreviations

The following abbreviations are used in this manuscript:

ALL	Acute Lymphoblastic Leukemia
ASIRw	Age-Standardized Incidence Rate
NCI	National Cancer Institute
CTCAE v5.0	Common Terminology Criteria for Adverse Events version 5.0
OS	Overall Survival
UTR	Untranslated Region
SNV	Single-Nucleotide Variant
INDEL	Insertion/Deletion
HWE	Hardy–Weinberg Equilibrium
OR	Odds Ratio
CI	Confidence Interval
TRM	Treatment-Related Mortality
NGS	Next-Generation Sequencing
BFM	Berlin–Frankfurt–Münster
TSER	Thymidylate Synthase Enhancer Region
