# Supplementary material for: Exploratory Association of 5′ and 3′ UTRs Variants in TYMS and CCND1 with Severe Hematologic Toxicity in Pediatric Acute Lymphoblastic Leukemia
Source: Int J Mol Sci. 2026 Sep 12;27(18):8131. doi: 10.3390/ijms27188131 (PMC13607164; doi:10.3390/ijms27188131)
Supplement: Supplementary file 1 [file ijms-27-08131-s001.zip › Supplementary figures.pdf]

Supplementary figures

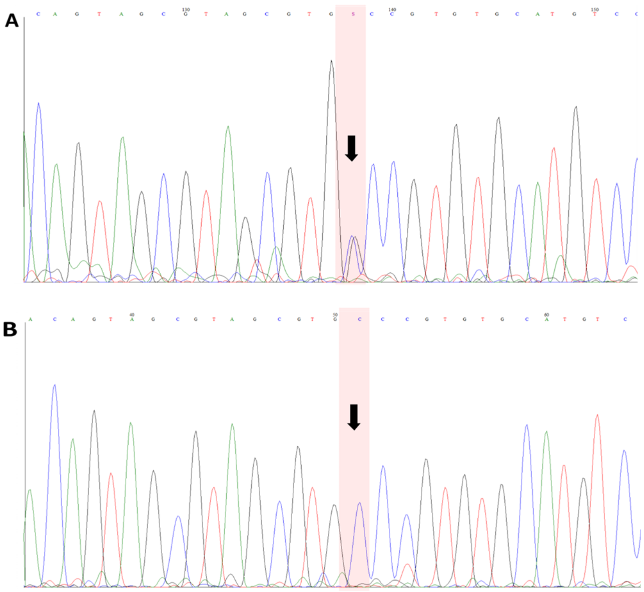

Supplementary Figure S1. Sanger sequencing analysis of the *CCND1* rs678653 variant (NM_053056.3:c.*687C>G) was conducted by direct sequencing of DNA from two representative pediatric ALL patients. (A) Heterozygous C/G genotype: overlapping peaks at the polymorphic site (arrow), indicated by the "S" IUPAC ambiguity code. (B) Wild type CC genotype: a single peak corresponding to the reference C allele (arrow). The chromatograms confirm the presence of the nucleotide substitution at the 3' untranslated region of *CCND1*.

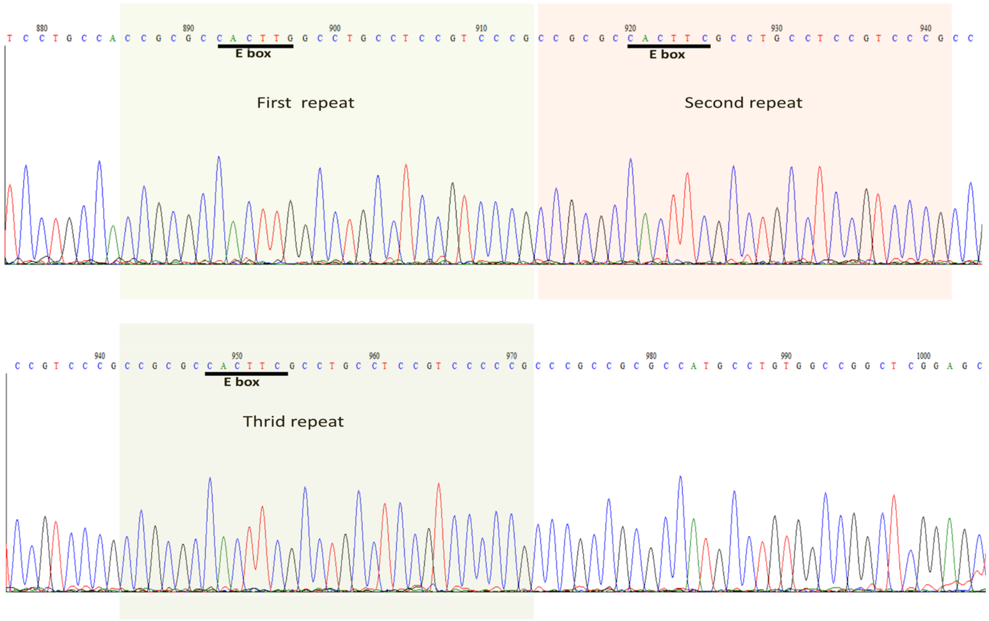

Supplementary Figure S2. Sequence analysis of the 5′ UTR of the thymidylate synthase gene (*TYMS*) contains three tandem repeats (3R) of 28-bp sequences. The E-boxes are located inside the repeat of the 3R allele.

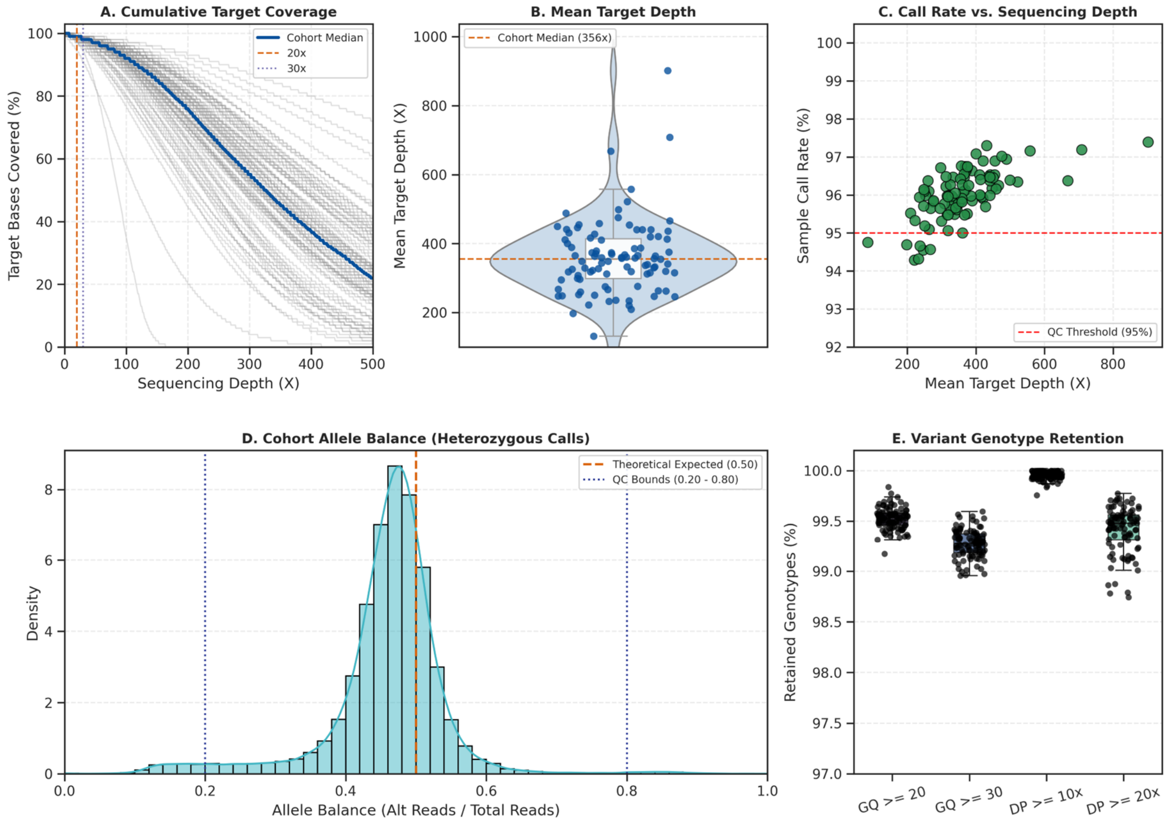

Supplementary Figure S3. Comprehensive sequencing quality control metrics across 96 targeted exome samples. (A) Cumulative target coverage showing the fraction of targeted bases (1,293,636 bp) covered at increasing depth thresholds. Grey curves: individual samples; blue curve: cohort median. Dashed vertical lines indicate ≥20× (>98.8%) and ≥30× (>97.5%) benchmarks. (B) Distribution Distribution of per-sample mean target depth (violin + boxplot + jitter), with cohort median >360× (IQR: 290× – 430×). (C) Genotype call rate vs. mean target depth. Red dashed line: 95.0% acceptance threshold. All samples exceeded a ≥94.5% call rate. (D) Allele balance (AB) distribution for all heterozygous calls, centered at 0.48, with >97.1% of calls within the 0.20–0.80 high-confidence window (dotted lines). (E) Genotype retention under increasing quality (GQ ≥20, GQ ≥30) and depth (DP ≥10×, DP ≥20×) filters, showing >99.8% retention across all thresholds.
